# Unconventional two-step spin relaxation dynamics of [Re(CO)_3_(im)(phen)]^+^ in aqueous solution†
†Electronic supplementary information (ESI) available: Computational details (initial conditions, electronic structure, excited-state dynamics, frozen-nuclei dynamics), data analysis details (populations, densities of states, emission spectrum, charge transfer analysis), verification of perturbational treatment of spin–orbit couplings compared to a variational treatment, electronic populations plot, population flux analysis, and analysis of luminescence decay contributions. See DOI: 10.1039/c9sc03671g


**DOI:** 10.1039/c9sc03671g

**Published:** 2019-09-27

**Authors:** Sebastian Mai, Leticia González

**Affiliations:** a Institute of Theoretical Chemistry , Faculty of Chemistry , University of Vienna , Währinger Straße 17 , 1090 Vienna , Austria . Email: leticia.gonzalez@univie.ac.at

## Abstract

Changes of molecular spin are ubiquitous in chemistry and biology. Among spin flip processes, one of the fastest is intersystem crossing (ISC) in transition metal complexes. Here, we investigate the spin relaxation dynamics and emission spectrum of [Re(CO)_3_(im)(phen)]^+^ (im = imidazole, phen = phenanthroline) using extensive full-dimensional excited-state dynamics simulations in explicit aqueous solution. Contrary to what has been observed in other transition metal complexes, the transition from the singlet to triplet states occurs *via* a two-step process, with clearly separable electronic and nuclear-driven components with two different time scales. The initially excited electronic wave function is a “molecular spin–orbit wave packet” that evolves almost instantaneously, with an 8 fs time constant, into an approximate 25 : 75 singlet-to-triplet equilibrium. Surprisingly, this ISC process is an order of magnitude faster than it was previously documented for this and other rhenium(i) carbonyl diimine complexes from emission spectra. Simulations including explicit laser field interactions evidence that few-cycle UV laser pulses are required to follow the creation and evolution of such molecular spin–orbit wave packets. The analysis of the dynamics also reveals a *retarded* ISC component, with a time constant of 420 fs, which can be explained invoking intramolecular vibrational energy redistribution. The emission spectrum is shown to be characterized by ISC convoluted with internal conversion and vibrational relaxation. These results provide fundamental understanding of ultrafast intersystem crossing in transition metal complexes.

## Abstract 2

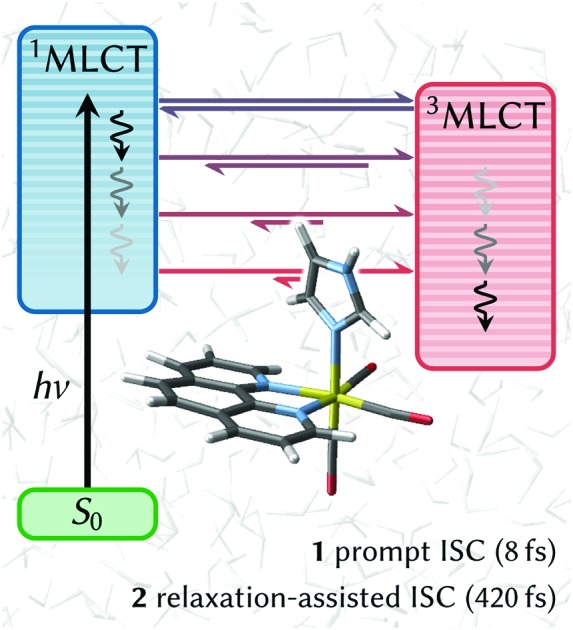
Full-dimensional excited-state dynamics simulations including explicit solvation show an unprecedented two-step intersystem crossing mechanism with electronic- and nuclear-driven components in [Re(CO)_3_(imidazole)(phenanthroline)]^+^.

## 1 Introduction

The phenomenon of molecular spin flip in transition metal complexes offers well-known prospects for applications in diverse areas extending from chemistry to biology.^1^–^3^ Especially non-radiative transitions between states with different spin have spurred a lot of research aimed at determining the involved time scales, intermediate species, as well as the often controversial involved spin relaxation mechanisms.^4^–^9^ In the past years, it has become apparent that the nonradiative change of electronic spin, *i.e.*, the so-called intersystem crossing (ISC),^2^,^10^ is not only controlled by the large spin–orbit coupling of the coordinating metal, but also by the complicated interplay of the high density of singlet and triplet states as well as steric and electronic effects from the surrounding ligands.^11^,^12^ As an example, ultrafast (10–50 fs) ISC have been measured in some mononuclear metal complexes—with [Ru(bpy)_3_]^2+^ and [Fe(bpy)_3_]^2+^ (bpy = 2,2′-bipyridine) providing two of the most compelling examples^4^,^5^,^9^—whereas much longer (picosecond) time scales are found in other complexes,^13^ without it being yet clear how these differences arise.^11^

Rhenium(i) carbonyl diimine complexes—convenient photosensitizers for studying long-range electron-transfer in proteins^14^,^15^—are another example of complexes where the detailed ISC mechanism remains elusive. Specifically, ISC, which facilitates a transition from the initially excited singlet metal-to-ligand charge transfer (^1^MLCT) states^16^–^18^ to several triplet states^16^,^19^–^24^ is reported on a 100 fs time scale, and thus slower than in other complexes containing Ru or Fe.

Understanding the factors that modulate and control ISC in molecules, also in the presence of an environment, is a considerable challenge, as the involved electronic states, *e.g.*, singlets and triplet species, can be very short-lived and difficult to detect experimentally. Theory, in contrast, is ideally suited to disentangle the character of the electronic states involved and their ultrafast non-radiative dynamics. However, for transition metal complexes, nonadiabatic dynamics studies in full dimensionality, which can unequivocally identify relaxation pathways and assign time constants, are only starting to emerge.^25^–^30^ Besides the breakdown of the Born–Oppenheimer approximation—leading to the appearance of strong nonadiabatic effects—and the explicit necessity of spin–orbit coupling, dealing theoretically with transition metal complexes entails the difficulty of considering a high density of electronic states and the complex interactions between the many electronic and nuclear degrees of freedom. Thus, most simulations dealing with transition metal complexes are restricted to few degrees of freedom, invoke approximations in the Hamiltonian considered, and/or do not include explicit solvation.

In this paper, we report the first full-dimensional nonadiabatic simulation of ISC dynamics in explicit aqueous solution of the rhenium carbonyl diimine complex [Re(CO)_3_(im)(phen)]^+^ (im = imidazole, phen = phenanthroline) and the simulation of its emission spectrum. The fact that Re has a larger spin–orbit coupling than Ru and Fe, and nevertheless Re(i) complexes displays a slower ISC rate than related Ru and Fe ones, is intriguing. Herein, we show that the process of ISC in [Re(CO)_3_(im)(phen)]^+^ is more complicated than anticipated and assigned experimentally.^23^ Our simulations reveal that ISC actually consists of two steps of different physical origin with associated different time constants: one particularly ultrafast on par with the large spin–orbit coupling of the Re atom and another longer one, that relates to vibrational relaxation within the coordination sphere. Furthermore, simulations including explicit laser fields illustrate that few-cycle laser pulses are required to resolve the few-fs explicit transition from singlet to triplet states in this kind of transition metal complexes. In contrast, the available measured^23^ emission spectrum is a simultaneous convolution of ISC, internal conversion, and vibrational energy redistribution.

## 2 Computational methods

The nonadiabatic dynamical simulations are performed with the SHARC (surface hopping including arbitrary couplings) approach^31^,^32^ in combination with a locally developed quantum mechanics/molecular mechanics (QM/MM) electrostatic embedding framework.^18^ Besides field–dipole interactions, the SHARC method takes into account nonadiabatic couplings and spin–orbit couplings “on-the-fly” and can thus describe non-trivial internal conversion and ISC dynamics simultaneously.

Details of the employed computational methods are given below as well as in Sections S1–S3 and Fig. S1–S4 in the ESI.†

### 2.1 Excited-state dynamics

Initial conditions to carry out dynamical simulations were generated from 500 snapshots of classical molecular dynamics (MD) simulations described elsewhere,^33^ where [Re(CO)_3_(im)(phen)]^+^ is solvated in a 12 Å truncated octahedron box of water plus a chloride ion. In the QM/MM procedure, only the Re complex is described quantum-mechanically by time-dependent density functional theory (TD-DFT), while the water molecules and chloride ion are in the MM region. We employ the Tamm–Dancoff approximation (TDA),^34^ with the B3LYP functional^35^ and dispersion correction,^36^ and the ZORA relativistic Hamiltonian,^37^ as implemented in the ADF2017 package.^38^ A mixed-ζ basis set was employed:^33^ ZORA-TZP^39^ for Re, and ZORA-DZP or ZORA-DZ for all other atoms. A perturbational ZORA formalism^40^ is employed to compute spin–orbit couplings. Mixed-quality integration grids were used, as described in ref. 33.

The electronic states that will be initially excited were selected stochastically based on the excitation energies and oscillator strengths in the excitation window of 2.8 eV to 3.2 eV, which corresponds approximately to the experimental excitation wave length of 400 nm.^23^ This energy range covers seven singlet states and eight triplet states; therefore, the simulations included a total of (7 + 8 × 3) 31 spin-mixed states. 151 initial conditions were accepted as follows: 43 in S_1_ (28%), 53 in S_2_ (35%), 25 in S_3_ (17%), 25 in S_4_ (17%), and 5 in S_5_ (3%). Out of these, 100 were simulated, and 94 were analyzed (6 trajectories were corrupted by network errors); from these, 29 started in S_1_ (31%), 32 in S_2_ (34%), 17 in S_3_ (18%), 14 in S_4_ (15%), and 2 in S_5_ (2%).

The excited-state dynamics simulations were carried out with the SHARC2.0 suite.^32^ The spin-mixed gradients were mixed^31^ from gradients of all singlets and triplets closer than 0.3 eV to the active state. The simulations were run until 250 fs, with a nuclear time step of 0.5 fs. The electronic wave function was propagated with a 0.02 fs time step with the local diabatization algorithm.^41^ Nonadiabatic couplings were obtained from wave function overlaps computed with the WFoverlap code,^42^ based on auxiliary wave functions generated from the TDA transition density matrix^28^ and truncated^42^ to 99.97% of the norm. During surface hops, the velocity vectors of all atoms of [Re(CO)_3_(im)(phen)]^+^ were rescaled, but not the velocities of MM atoms. Similarly, in the energy-based decoherence correction^43^ instead of the full kinetic energy of the system we only considered the kinetic energy of the [Re(CO)_3_(im)(phen)]^+^ atoms.

Frozen-nuclei dynamics simulations were also performed using the same settings, except that nuclear motion was set to zero, and the quantum-chemical data from the first time step of each trajectory was reused in all time steps. In this case, 94 trajectories were propagated until 50 fs.

### 2.2 Data analysis

**Fig. 1:**
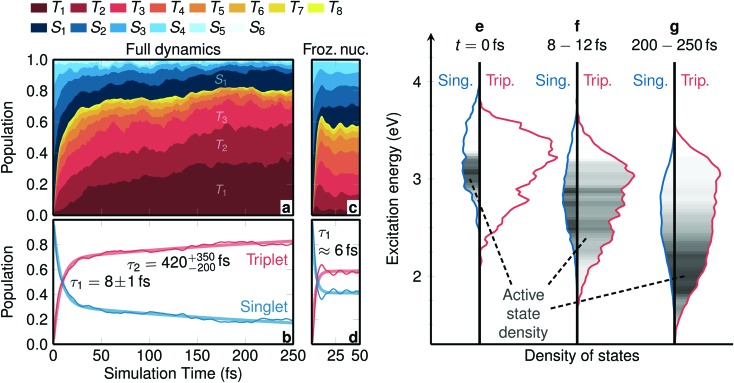
Temporal evolution of electronic populations and of densities of states. (a) Electronic populations calculated at the TD-B3LYP/T-DZP level of theory, see Fig. S5† for a regular line plot. (b) Total singlet and triplet populations (thin lines) and biexponential fit (thick lines). The labels give the fitted ISC time constants and their errors from bootstrapping. (c and d) Same data as in (a and b), but considering frozen nuclei. (e–g) Singlet and triplet densities of states at *t* = 0 fs, averaged between *t* = 8 fs and *t* = 12 fs, and averaged between *t* = 200 fs and *t* = 250 fs. The grey shading within the plots depicts the density of the active state of the trajectories.

To ease interpretation, the spin-mixed coefficients from the SHARC simulations were transformed into the spin-free populations from Fig. 1 as: 
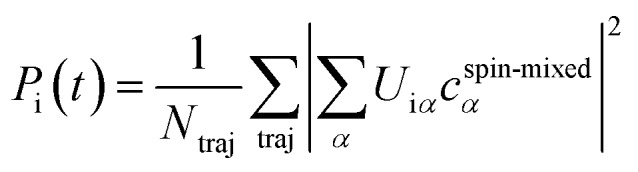
, with the spin-free–spin-mixed transformation matrix **U**. The populations were fitted with the function *f*(*t*) = *R*_fast_e^–*t*/*τ*_fast_^ + (1 – *R*_fast_)e^–*t*/*τ*_slow_^ and errors were computed by bootstrapping. The densities of state in Fig. 1 are convolutions (FWHM of 0.05 eV) of all energy differences between the S_0_ and the singlet, triplet, or active states. The simulated emission spectrum is a two-dimensional convolution of the active–ground state energy differences and the oscillator strengths, with 0.25 eV × 100 fs Gaussians. The character of the involved electronic excited states was analysed with a charge transfer analysis carried out with TheoDORE,^44^,^45^ where charge transfer numbers were weighted with the electronic populations and averaged over all trajectories.

### 2.3 Frozen-nuclei dynamics with explicit laser fields

Simulations using explicit laser fields were carried out for one frozen-nuclei trajectory. The laser pulses used a randomly chosen polarization vector, a phase of 0, a central energy of 3.3397 eV (corresponding to the energy of a bright spin-mixed state for that geometry), and a sin^2^ envelope. The maximum field strengths were chosen to transfer about 75% of the ground state population to the excited states. Only the transition dipole moments between S_0_ and the excited singlet states were considered.

## 3 Results and discussion

The temporal evolution of the spin-free, electronic populations after excitation is shown as a stacked plot in Fig. 1a (see also Fig. S5 in Section S4†). Initially, the population is mainly distributed over the S_1_ to S_4_ singlet states, with no population in the triplet states. This is due to the fact that before excitation the electronic wave function is a pure singlet ground state (total spin expectation value of singlet states, with no population in the triplet states. This is due to the fact that before excitation the electronic wave function is a pure singlet ground state (total spin expectation value of 〈*ŝ*^2^〉 ≈ 0.002), and the instantaneous vertical excitation (equivalent to a δ-laser pulse) does not change the spin expectation value. However, after vertical excitation the electronic wave function is not anymore an eigenstate of the total Hamiltonian; due to the strong spin–orbit couplings it can be best described as a linear combination of spin–orbit eigenstates. Such a linear combination—that might be called a “molecular spin–orbit wave packet” (see Section S5 ≈ 0.002), and the instantaneous vertical excitation (equivalent to a δ-laser pulse) does not change the spin expectation value. However, after vertical excitation the electronic wave function is not anymore an eigenstate of the total Hamiltonian; due to the strong spin–orbit couplings it can be best described as a linear combination of spin–orbit eigenstates. Such a linear combination—that might be called a “molecular spin–orbit wave packet” (see Section S5† for a definition)—undergoes nontrivial time evolution, which manifests itself in ISC that occurs almost instantaneously. Within 30 fs the triplet population grows to 70%, whereas at later times the triplet population grows much slower, reaching *ca.* 80% after 250 fs.

The different population behavior before and after 30 fs hints at a two-step process. Indeed, the total singlet and triplet populations are best fitted with a biexponential model from which two time constants are obtained, see Fig. 1b. We shall label the time constant *τ*_1_ = 8 ± 1 fs as “*prompt* ISC” and *τ*_2_ = 420+350–200 fs as “*retarded* ISC”. The *prompt* ISC has a fitted contribution of 69% ± 9%. On a side note, Fig. 1a also illustrates that ISC occurs before the higher singlet states are depopulated. This is a direct demonstration that ISC in [Re(CO)_3_(im)(phen)]^+^ contradicts (see also Fig. S6†) the common assumption that a photoexcited system first decays to the lowest excited state of the initial multiplicity, before any other processes (emission, ISC) occur. In [Re(CO)_3_(im)(phen)]^+^, ISC does not primarily (<25%) occur from the lowest S_1_ state, similar to what we found^28^ in [Ru(bipy)_3_]^2+^ and has been postulated in other complexes.^46^

To disentangle the different contributions to ISC, we first investigate the time evolution of the electronic wave function decoupled from nuclear motion, using SHARC dynamics simulations with the full initial distribution of geometries but freezing nuclear motion. The resulting, specific and summed-up, electronic populations of the frozen dynamics are shown in Fig. 1c and d, respectively. As in the dynamic simulations including nuclear relaxation, initially only singlet states are populated, but within only 20 fs about 60% of the population is promptly transferred into the triplet states. This effect is due to the evolution of the spin–orbit wave packet, which involves periodic population transfer, like Rabi oscillations, between the strongly spin-coupled singlets and triplets, but with the different trajectories quickly dephasing due to the variation of the energy gaps across the distribution of nuclear geometries. The fact that *prompt* ISC is present in these simulations, despite the nuclear motion being frozen, evidences that this is a purely electronic effect.

In contrast to the purely electronic *prompt* ISC, the *retarded* ISC component with *τ*_2_ = 420+350–200 fs can only be understood by considering nuclear relaxation. Fig. 1e–g show the singlet and triplet densities of states as well as the density of the active surface hopping state for three representative time intervals. At *t* = 0 fs, the singlet states S_1_ to S_6_ are primarily distributed between 2.8 eV and 3.7 eV and the triplet states T_1_ to T_8_ between 2.5 eV and 3.6 eV. According to the chosen excitation window, the active state is located between 2.8 eV and 3.2 eV within the singlet manifold. In this energy range, the triplet density is approximately three times as large as the singlet one, due to the three M_S_ components of the triplet. At around *t* = 10 fs, nuclear relaxation has begun to shift all electronic states to lower energies and activate nonadiabatic transitions (IC) that move the active state density down. On the same time scale, the extremely efficient *prompt* ISC quickly establishes a statistical distribution with an approximately 25 : 75 (singlet : triplet) ratio. This basically reflects that ISC is so fast that the system rapidly finds a dynamic equilibrium between singlet and triplet states, where the point of equilibrium is controlled by the densities of states. At later times, as the system continues vibrationally relaxing to lower energies, eventually there is less and less singlet state density available, forcing the overall spin density to become more and more triplet-like. It is precisely this process that is responsible for the *retarded* temporal ISC component visible in Fig. 1b. Very likely this intramolecular vibrational energy redistribution will continue after 250 fs, presumably reaching an almost pure triplet spin after a few ps.

It is possible to compare the evolution of the total singlet and triplet populations with that obtained by Fumanal *et al.*^47^ using quantum dynamics simulations including 15 harmonic normal modes but no explicit solvent motion. Unfortunately, these authors do not discuss in detail the ultrafast decay of the initial singlet population discernible in their Fig. 3 (left).^47^ In the light of our findings, we would attribute this decay to the *prompt* ISC. The fact that both simulations find this ultrafast initial decay is encouraging, showing that very distinct treatments of nuclear motion (quantum-mechanical *vs.* surface hopping, see also ref. 48) do not affect this purely electronic ISC process. However, for later times it is not possible to clearly identify a decay of the singlet population in ref. 47. Hence, we refrain from making any comparison regarding our *retarded* ISC component.

**Fig. 2:**
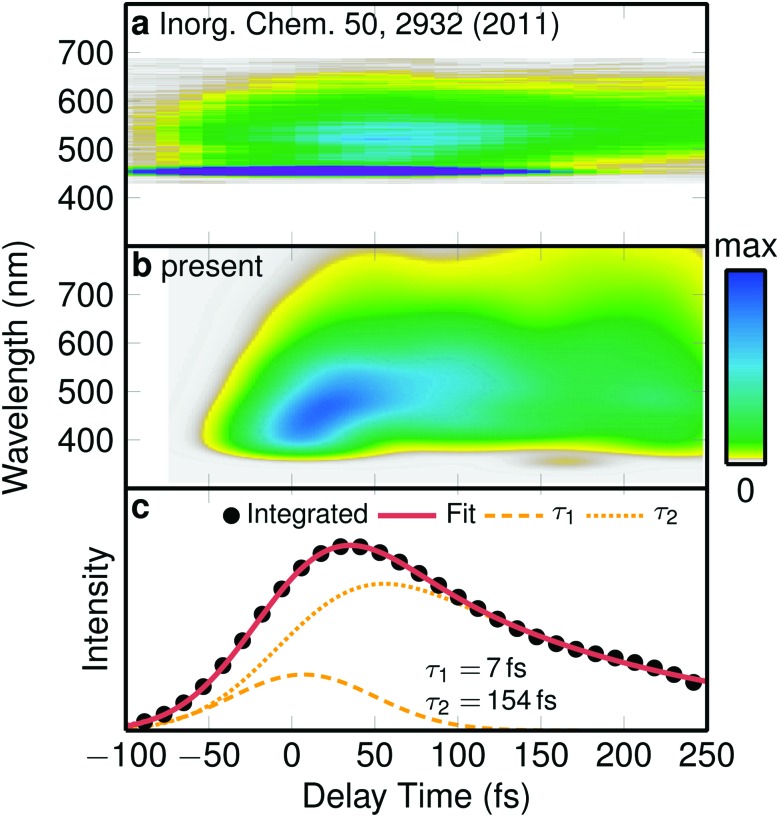
Emission spectrum. (a) Experimental time-resolved emission spectrum.^23^ (b) Simulated emission spectrum, convoluted with a 0.25 eV × 100 fs Gaussian. (c) Integrated simulated spectrum and biexponential fit with time constants given by the labels.

**Fig. 3:**
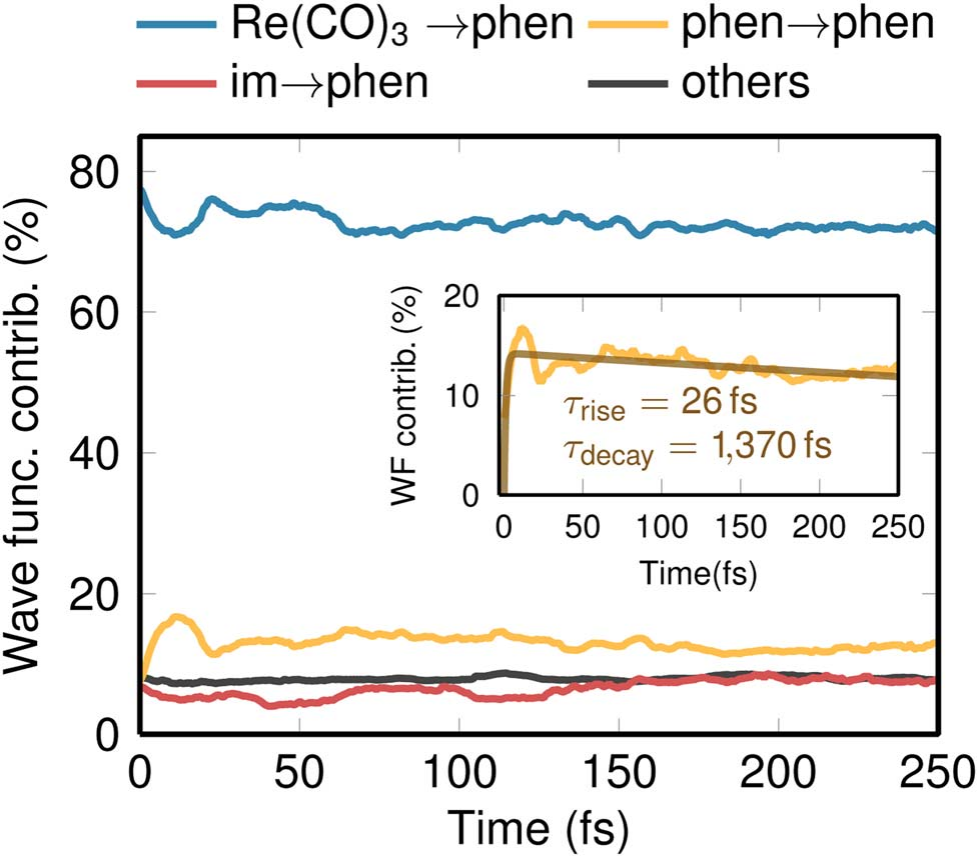
Temporal evolution of the total electronic wave function composition. The different charge-transfer and local transitions were obtained through partitioning of the transition density matrices and subsequent weighting with the populations and averaging over all trajectories. The Re(CO)_3_ → phen contribution corresponds to singlet and triplet MLCT, the phen → phen contribution to ^3^IL states, the im → phen contributions to LLCT states. The inset shows an enlarged plot of the phen → phen contribution, with a biexponential fit with *τ*_rise_ = 26 fs and *τ*_decay_ = 1370 fs.

Intriguingly, neither the *prompt* nor the *retarded* ISC time constants obtained with SHARC in solution agrees with the time constants extracted from time-resolved experiments on a number of Re(i) complexes from the last decade.^16^,^20^,^22^–^24^ Depending on the ligands and the experimental setup, transient absorption and fluorescence up-conversion experiments observed decay times between 80 fs and 150 fs. These time constants were assigned to ISC, and hence it was presumed that ISC in Re(i) carbonyl complexes was anomalously slower than in Ru or Fe complexes and do not scale with the spin–orbit coupling of the metal center.^16^,^20^

Puzzled by the discrepancy between the experimentally assigned time constant for ISC in [Re(CO)_3_(im)(phen)]^+^ (144 fs)^23^ and our simulated ones (8 fs and 420 fs), we decided to simulate the same observable as in the experiment, *i.e.*, we calculated a time-resolved emission spectrum from our trajectory data, temporally convoluted with a 100 fs Gaussian corresponding to the experiments.^23^ The experimental spectrum,^23^ shown in Fig. 2a, starts with a maximum at about 520 nm and evolves to a weaker signal at 560 nm that decays within a few hundred fs. The simulated one (Fig. 2b) starts around 450 nm, quickly shifts to about 500 nm, and subsequently decays. Despite the computed spectrum being slightly broader energetically than the experimental one, the temporal profiles of both spectra agree satisfactory with each other. In order to obtain a time scale of the decay of the simulated emission, we integrated the spectrum and fitted it to a temporally broadened bi-exponential function (Fig. 2c). The fit yielded two time constants of *τ*em1 = 7 fs and *τ*em2 = 154 fs. While *τ*em1 is below the experimental time resolution, *τ*em2 is in excellent agreement with the experimental value of 144 fs,^23^ confirming the reliability of our simulations.

One important conclusion of this finding is to realize that the simulated emission time *τ*em2 = 154 fs does not match either of the two ISC time constants (*τ*_1_ = 8 fs and *τ*_2_ = 420 fs) that were obtained from the population data. Thus, the experimentally observed luminescence decay should not be ascribed directly to ISC. Instead, luminescence decay is the result of a complicated combination of ISC, reduction of transition dipole moments due to nuclear motion, and internal conversion from brighter states to darker states (illustrated in Fig. S7†). As a corollary, these findings show that the ultrafast component of 8 fs in [Re(CO)_3_(im)(phen)]^+^ rivals the ISC time scales of [Ru(bpy)_3_]^2+^ or [Fe(bpy)_3_]^2+^ complexes,^4^,^5^,^9^ in agreement with the larger spin–orbit coupling of the Re metal.

Another important gain of our spin relaxation dynamics study is the elucidation of the nature of the electronic states involved. Decomposing the total electronic wave function in terms of charge transfer contributions,^44^,^45^ it is possible to follow the character of the wavefunction in time, as shown in Fig. 3. According to literature,^16^–^18^ the initially excited low-lying singlet states of Re(i) carbonyl diimine complexes have predominant MLCT character—or more precisely a mixture of MLCT and LLCT, which we denote as “Re(CO)_3_ → phen”. As Fig. 3 shows, such states contribute about 80% at *t* = 0 with minor contributions coming from IL states (phen → phen), LLCT (im → phen) states, and additional small contributions from molecular orbitals that are delocalized over all ligands. One issue intensely discussed in the literature is the role of the IL states.^16^,^20^,^23^ Experimentally, ^3^IL states were assigned to be populated by 11–20% and to decay to the lower ^3^MLCT states within 1–3 ps.^23^ Pleasingly, our simulations nicely reproduce this trend (see inset in Fig. 3). A bi-exponential fit of the ^3^IL time evolutions gives a rise time of about 26 fs (slightly slower than *prompt* ISC), a maximum population of 17%, and a decay time constant of 1370 fs.

**Fig. 4:**
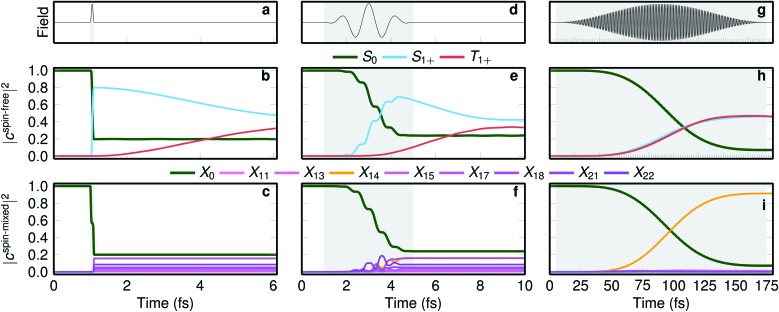
Laser excitation simulations with different pulse durations. The top row shows three laser pulses with full width at half maximum (FWHM) of (a) 0.1 fs, (d) 2 fs, and (g) 85 fs. (b, e, h) The middle row shows the laser-driven populations in the spin-free basis, and (c, f, i) the bottom row the populations in the spin-mixed basis. The grey area denotes the time window where the pulse is on.

At this point it is fair to wonder how the dynamics simulations, which assume a δ-pulse excitation, correlate with experiments with finite-duration laser pulses. To answer this question, we have carried out three simulations with frozen nuclei including explicit laser pulses of different durations, from a width of 0.1 fs (an approximate δ-pulse) to 85 fs (similar to the experimental setup of ref. 23). Fig. 4 shows the laser fields (top panels), the corresponding evolution of the spin-free states (mid panels) where singlet and triplet can be distinguished and thus laser excitation is discriminated from ISC, and of the spin-mixed states (lower panels). Although the spin-free and spin-mixed representations are equivalent quantum-mechanically, their interpretation is not equally intuitive.

The δ-pulse excitation (Fig. 4a–c) excites about 80% population from the ground state to the singlet states but zero to the triplets due to the selection rules that forbid direct singlet–triplet excitations. Triplet population rises when the pulse is already over, as visible in Fig. 4b. Fig. 4c shows that the action of the laser pulse is to form a linear combination of eigenstates of the spin–orbit Hamiltonian, with coefficients such that the total wave function is a pure singlet. This linear combination is the spin–orbit wave packet discussed above. In simple terms, the very short pulse excites the wave function vertically, and because the ground state wave function is a pure singlet, the vertically excited wave packet must be also a pure singlet. After the pulse is over, the spin-mixed populations |*c*spin-mixedi|^2^ do not change anymore (Fig. 4c), but the complex coefficients *c*spin-mixed*i* do evolve in time, making the singlet and triplet populations (Fig. 4b) change with time.

When a few-cycle pulse with FWHM of 2 fs is employed (see Fig. 4d–f), still only singlet states are directly excited but within the duration of the pulse, the triplet population starts growing. As in the δ-pulse, the spin-mixed populations of Fig. 4f show the formation of a spin–orbit wave packet. Interestingly, when a long laser pulse (FWHM of 85 fs) is applied (Fig. 4g–i) a concerted growth of singlet and triplet populations is apparent (Fig. 4h) but no spin–orbit wave packet is formed, as only one spin–orbit eigenstate is significantly populated (Fig. 4i). As this pulse is much longer than the rapid exchange between singlets and triplets, such a pulse does not allow identifying the time scale of ISC. Stated differently, while a long, monochromatic laser pulse allows exciting to a single eigenstate, in transition metal complexes with large spin–orbit coupling this state will be so heavily spin-mixed that it is not possible to observe ISC, *i.e.*, a change from a singlet to a triplet.

Fig. 4 therefore suggests that very short laser pulses are necessary to observe the few-fs *prompt* ISC that occurs in some transition metal complexes. With the longer laser pulses currently employed in many experiments (tens of fs, *e.g.*, as in ref. 23), ISC takes place already within the pulse duration, so that excitation and ISC cannot be discriminated, and ISC cannot be independently observed. We note here, however, that our simulated emission spectrum in Fig. 2 can still be compared to the experimental one due to the applied temporal convolution, which matches our time resolution to the experimental one.

## 4 Conclusion

Contrary to the conception that nuclear relaxation is needed to drive ISC, the herein presented simulations on [Re(CO)_3_(im)(phen)]^+^ evidence a two-step ISC process—resulting from the interaction of extremely efficient, electronically-driven ISC and nuclear relaxation—with time constants of 8 fs and 420 fs, respectively. The ultrafast ISC component quickly establishes a dynamical equilibrium between singlet and triplet states in a 25 : 75 ratio, whereas nuclear relaxation shifts the singlet–triplet ratio towards a pure triplet state on a slower time scale. These results will allow for new interpretations of spin relaxation phenomena in transition metal complexes—for example in terms of spin–orbit wave packets—which are key to design photonic materials of current interest. To observe the presented ultrafast few-fs ISC experimentally, very short—few-cycle—ultraviolet laser pulses are necessary, as the longer pulses typically employed currently (*e.g.*, FWHM ≈ 85 fs (ref. 23) in [Re(CO)_3_(im)(phen)]^+^) are longer than the equilibration between singlet and triplet states takes, making singlet–triplet transitions unobservable. This work also showcases the potential of current computational technologies to unravel spin relaxation and vibrational coupled dynamics in systems with strong relativistic effects.

## Conflicts of interest

There are no conflicts to declare.

## Author contributions

SM carried out all parts of the simulations and data analysis, and wrote the initial manuscript draft. LG and SM conceptualized the research, interpreted the results, and wrote the final manuscript.

## Supplementary Material

Supplementary information
