# Supplementary material for: Unconventional two-step spin relaxation dynamics of [Re(CO)3(im)(phen)]+ in aqueous solution
Source: Chem Sci. 2019 Sep 27;10(44):10405–11. doi: 10.1039/c9sc03671g (PMC6988600; doi:10.1039/c9sc03671g)
Supplement: Supplementary file 1 [file SC-010-C9SC03671G-s001.pdf]

Electronic Supplementary Material (ESI) for Chemical Science.
This journal is © The Royal Society of Chemistry 2019

Electronic Supplementary Information:

Unconventional two-step spin relaxation dynamics in a rhenium complex

Sebastian Mai[1] and Leticia González[1,*]

[1] Institute of Theoretical Chemistry, University of Vienna, Währinger Straße 17, 1090 Vienna, Austria.

[*] leticia.gonzalez@univie.ac.at

Contents

S1 Supplementary methods: Computational details **2**
- S1.1 Initial condition generation 2
- S1.2 Electronic structure 3
- S1.3 SHARC excited-state dynamics 4
- S1.4 SHARC frozen-nuclei dynamics 4
- S1.5 Frozen-nuclei dynamics with explicit laser fields 4

S2 Supplementary methods: Data analysis **6**
- S2.1 Electronic populations and representations 6
- S2.2 Densities of states 6
- S2.3 Time-resolved emission spectrum 6
- S2.4 Charge-transfer character analysis 7

S3 Supplementary results: Perturbational spin-orbit couplings **9**

S4 Supplementary results: Evolution of electronic populations **11**

S5 Supplementary results: Definition of the spin–orbit wave packet **12**

S6 Supplementary results: Non-Kasha behavior in population flux **14**

S7 Supplementary results: Luminescence decay **15**

S1 Supplementary methods: Computational details

The excited-state dynamics simulations consisted of three main steps: (i) sampling of the nuclear phase space in the electronic ground state to provide appropriate initial conditions,[1] the SHARC dynamics simulations, and data analysis. Unless otherwise noted, classical MD simulations were performed with AMBER16,[2] electronic structure and QM/MM calculations with a development version of ADF2017,[3] and the dynamics simulations itself with SHARC2.0.[4,5]

S1.1 Initial condition generation

The procedure of sampling the ground state coordinates and velocities is reported elsewhere,[1] and only briefly presented here. Supplementary Fig. S1 gives a graphical overview over the steps involved.

First, classical molecular dynamics simulations were carried out. The force field employed for the complex was taken from the generalized AMBER force field,[6,7] where the missing bonded parameters involving Re were parametrized based on a few-ps QM/MM trajectory. The partial charges were obtained from a RESP fit[8] to electron density obtained at the B3LYP/6-31G* (LANL2DZ for Re[9]) level of theory, using the Gaussian09 software.[10] The Van-der-Waals radius of Re was set to 1.47 Å.[11] The complex was solvated in a 12 Å truncated octahedron box of 1054 TIP3P[12] H_2O molecules and a chloride ion was added to neutralize the charge of the complex. After minimization, the system was thermalized to 300 K (NVT ensemble) for 20 ps, followed by equilibration to 1 bar for 200 ps (NPT ensemble). A 10 ns production run (NPT ensemble) was used to obtain 500 equally spaced geometry/velocity snapshots. These steps are represented by the horizontal arrows in Supplementary Fig. S1. All MD simulations employed a time step of 0.5 fs, which was necessary in order to avoid constraining the C–H and N–H bond vibrations of the complex. Note that the O–H bond lengths of the water molecules were also not constrained.

As discussed in Refs. 1,13–15 classical MD at 300 K will bestow too little internal energy to a molecule like $[Re(CO)_3(Im)(Phen)]^+$, compared to the zero-point energy. In order to provide a more appropriate amount of internal energy, each of the 500 snapshots was heated to 600 K for 20 ps with all solvent molecules frozen (blue arrows in Supplementary Fig. S1). Subsequently, all atoms were imaged into the

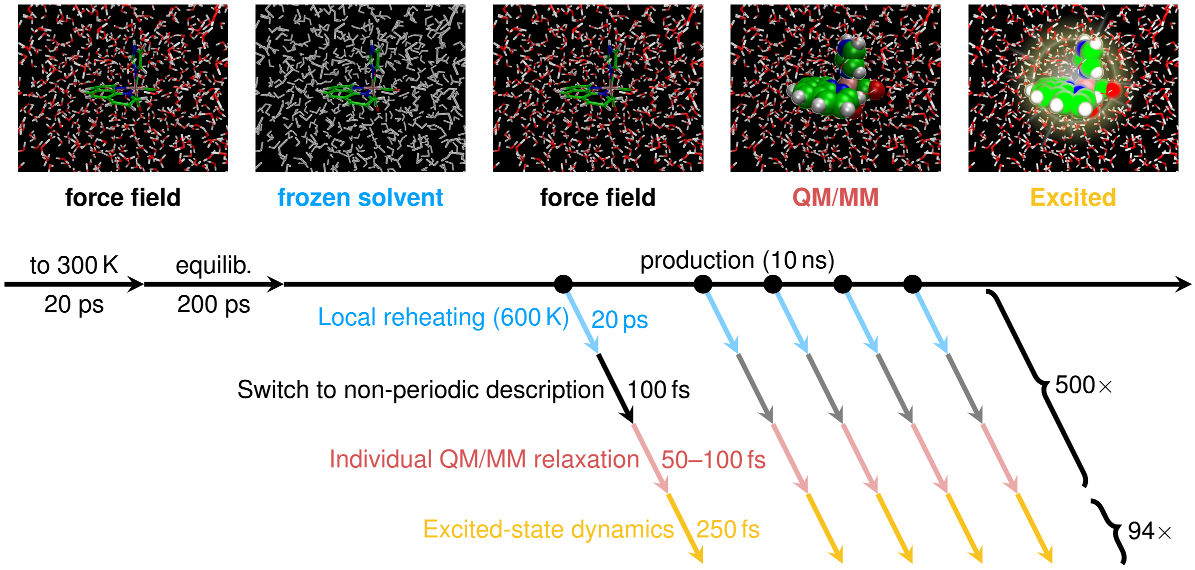

Figure S1: Preparation of initial conditions. The main steps were classical, force-field-based MD simulations to sample 500 molecular snapshots, local reheating of the solute, QM/MM relaxation in the ground state, and excited-state dynamics simulations. In the excited state, less trajectories were run, because not every initial condition was selected for excitation in the initial state selection scheme.

primary cell, periodic boundary conditions, barostat and thermostat turned off, and the system propagated for 100 fs in the NVE ensemble (black arrows in Supplementary Fig. S1). The resulting endpoints of the 500 trajectories were collected and converted into SHARC initial condition format. During this conversion, we accounted for the fact that AMBER uses a leapfrog-like algorithm (which stores coordinates $\vec{R}(t)$ and velocities $\vec{v}(t-\frac{\Delta t}{2})$) whereas SHARC uses the velocity Verlet algorithm (coordinates $\vec{R}(\tau)$ and velocities $\vec{v}(\tau)$). To compensate this, we computed

$$\vec{R}(\tau) = \vec{R}\left(t - \frac{\Delta t}{2}\right) = \vec{R}(t) - \frac{\Delta t}{2}\vec{v}\left(t - \frac{\Delta t}{2}\right), \tag{1}$$

where Δt was the time step employed in the AMBER simulations (0.5 fs).

Subsequently, we ran short ground state QM/MM trajectories (level of theory in Supplementary Section S1.2) in order to reduce any effects from imperfect force field parameters. According to Ref. 1, this short relaxation at QM/MM level shifts all bond length and angle distributions much closer to the ab initio optimized values, and shifts the absorption spectrum (see below) by 0.2 eV. In order to avoid that coherent motion originating from the switch from force field to ab initio is carried to the excited state, the short QM/MM trajectories were run for randomized simulation times (between 50 fs and 100 fs). The end points of these trajectories were then collected and constitute the actual starting points of the excited-state simulations.

Besides initial coordinates and velocities, the initial active state and electronic wave function coefficients are also required to launch the excited-state trajectories. These were obtained for each geometry from a vertical excitation calculation including 30 singlet states at the level of theory given in Supplementary Section S1.2. The absorption spectrum, obtained by summing over all 500 snapshots and convoluting with Gaussians (full width at half maximum (FWHM) of 0.1 eV), is shown in Supplementary Fig. S2. The most relevant part of the spectrum is located around the experimental excitation energy of $[Re(CO)_3(Im)(Phen)]^+$, which is 400 nm.[16] Vertical excitation at this wave length can populate the adiabatic states S_1 to S_5. Whether an initial condition is accepted and which state is actually the initial state for each of the 500 geometries was decided by a stochastic algorithm.[17] Here, in order to receive a sufficient number of initial states from the 500 geometries, we chose an excitation window of 2.8 eV to 3.2 eV, which contains the experimental excitation energy and only the states S_1 to S_5. Inside this window, the stochastic algorithm accepted 151 initial conditions, with 43 starting in S_1 (28%), 53 in S_2 (35%), 25 in S_3 (17%), 25 in S_4 (17%), and 5 in S_5 (3%). Out of these 151 initial conditions, 100 were actually simulated. From these, 6 were unusable due to network errors that lead to corrupted restart files. Hence, the results presented here and in the main text are based on 94 trajectories, out of which 29 started in S_1 (31%), 32 in S_2 (34%), 17 in S_3 (18%), 14 in S_4 (15%), and 2 in S_5 (2%). As can be seen no initial state was underrepresented in the final set of trajectories. The initial wave function coefficients were simply set to $c_i^{\text{spin-free}} = \delta_{i\alpha}$, where α is the initial active spin-free state.

S1.2 Electronic structure

All electronic structure calculations performed during the ground state QM/MM dynamics,[1] the vertical excitation calculations, and the excited-state dynamics simulations employed the same electronic structure level of theory: (time-dependent) B3LYP[18] with dispersion correction[19] and the ZORA relativistic Hamiltonian,[20] as implemented in the ADF2017 package[3]. A mixed-ζ basis set was employed for an optimal compromise between accuracy and efficiency:[1] ZORA-TZP[21] for Re, ZORA-DZ for H atoms and the four C/N atoms of the imidazole ligand not bonded to Re, and ZORA-DZP for all other atoms. No electrons were frozen and standard SCF settings were used. Solvation effects were considered through electrostatic embedding[22], where $[Re(CO)_3(Im)(Phen)]^+$ constituted the QM region, and the water

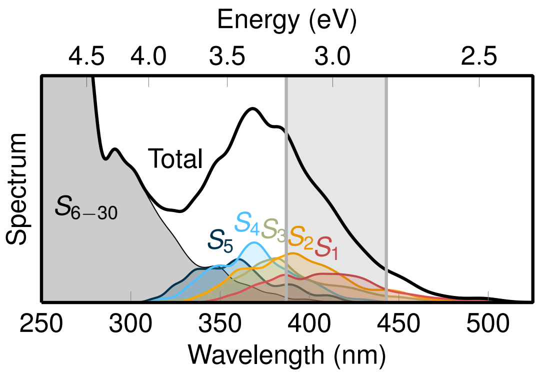

Figure S2: Simulated absorption spectrum of $[Re(CO)_3(Im)(Phen)]^+$ in aqueous solution. The total spectrum is given by the thick black line, the contributions of the individual spin-free adiabatic states are shown in color. The excitation energy window from where initial states were drawn is shown as a light grey box.

molecules and chloride ion the MM region. Excited states were computed with the Tamm-Dancoff approximation (TDA), and SOCs were computed through a perturbational ZORA formalism.[23] Mixed-quality integration grids were used.[1]

S1.3 SHARC excited-state dynamics

We considered 7 singlet states (ground state plus 6 excited singlets) and 8 triplet states, i.e., a total of 31 spin-mixed states (7+3×8). In order to construct the gradient of the current active spin-mixed state, we mixed[5] the gradients of all singlet and triplet states that are closer than 0.3 eV to the active state; in this way, we reduce the amount of gradient computations while keeping accurate spin-mixed gradients.

The simulations were run until 250 fs, with a nuclear time step of 0.5 fs. The electronic wave function was propagated with a 0.02 fs time step with the local diabatization algorithm.[24] The necessary wave function overlaps were computed with the WFoverlap code[25] based on auxiliary wave functions generated from the TDA transition density matrices.[26] In order to speed up these overlap calculations, the auxiliary wave function vectors were truncated[25] to 99.97% of the norm, keeping only the largest vector elements.

During surface hops, the velocity vectors of all atoms of $[Re(CO)_3(Im)(Phen)]^+$ were rescaled, but not the velocities of MM atoms. Similarly, in the energy-based decoherence correction[27] instead of the full kinetic energy of the system we only considered the kinetic energy of the $[Re(CO)_3(Im)(Phen)]^+$ atoms.

S1.4 SHARC frozen-nuclei dynamics

The frozen-nuclei dynamics was carried out with the same settings mentioned above, except that the nuclei were replaced by a single dummy atom and the nuclear velocity was set to zero (which also renders the decoherence correction inactive). The quantum-chemical data (Hamiltonian matrix, transition dipole moment matrices) from the first time step of each trajectory was fed to SHARC at all time steps. Gradients were set to zero and the overlap matrix to a unit matrix. In this way, 94 trajectories were propagated until 50 fs.

S1.5 Frozen-nuclei dynamics with explicit laser fields

The frozen-nuclei dynamics including explicit laser fields was done for a single frozen-nuclei trajectory, using three different laser fields. In all cases, the same randomly chosen polarization vector, a phase of 0, and a central energy of 3.3397 eV (corresponding to the energy of a bright spin-mixed state for that

geometry) were chosen. The envelope was of $\sin^2$ type: the laser was off for the first 1 fs, then the envelope increased between 1 fs and the pulse center as $\sin^2$, and then decreased over the same time span, giving a symmetric envelope. The simulations were continued for 5 fs after the pulse finished. The maximum field strengths were chosen to transfer about 75% of the ground state population to the excited states. Note that the simulations only considered the transition dipole moments between S_0 and the excited singlet states; all other static and transition dipole moments were omitted from the dipole moment matrices.

S2 Supplementary methods: Data analysis

S2.1 Electronic populations and representations

In SHARC, the actual nuclear dynamics is propagated in the so-called "diagonal" representation of the electronic states.[5] The diagonal states are computed as the eigenstates of the full molecular Hamiltonian including spin–orbit couplings (also called "spin-mixed states" in the main text). Because directly computing these eigenstates is very demanding, we apply a two-step procedure.[23,28] First, a number of relevant eigenstates of the (spin-free) molecular Coulomb Hamiltonian (MCH) are obtained as usual with electronic structure code (in this case, ADF), and subsequently the full Hamiltonian matrix in the basis of these MCH eigenstates is constructed (including the MCH eigenenergies and spin–orbit couplings) and diagonalized to produce the diagonal states. A validation of this two-step spin–orbit treatment is presented in Supplementary Section S3.

Due to the two-step procedure, in SHARC the MCH–diagonal transformation matrix is known and it is possible to either analyze the state populations in the diagonal or the MCH representation. The electronic populations in the MCH (i.e., spin-free) representation can be computed in two different ways. On one hand, one can compute "classical" populations, which requires that the active states in the diagonal representation of all trajectories and all time steps is mapped to MCH states, which can then be counted. This provides statements like "At time step t, 80% of trajectories are moving in active states that are predominantly S_2." However, due to strong mixing in the present case, this mapping is non-trivial. On the other hand, one can compute "quantum" populations by incoherent averaging over the quantum amplitudes. The latter can easily be transformed between diagonal and MCH representation:

$$P_i^{\mathrm{MCH}}(t) = \frac{1}{N_{\mathrm{traj}}} \sum_{\mathrm{traj}} \left| \sum_{\alpha} U_{i\alpha} c_{\alpha}^{\mathrm{diag}} \right|^2, \tag{2}$$

where i is an MCH state, α is a diagonal state, and $\mathbf{U}$ is the MCH–diagonal transformation matrix. Note that here we employ a decoherence correction that makes the classical and quantum populations approximately equal. We also note that we obtain identical results when computing the electronic populations with the quasi-Wigner protocol of Subotnik and coworkers,[29] showing that the "quantum" populations are appropriate.

We also note that the obtained MCH populations give no indication about the electronic character (i.e., they are not diabatic). The electronic character can be analyzed through the charge transfer analysis (see Supplementary Section S2.4).

S2.2 Densities of states

The densities of states were obtained by collecting all energy differences between the S_0 and the computed singlet and triplet states and convoluting this data with Gaussians (FWHM of 0.05 eV). The active state density was likewise obtained from the energy differences between S_0 and the current active spin-mixed state. For Figure 1f–g, these convoluted densities of state were averaged over the time intervals 8–12 fs (Figure 1f) and 200–250 fs (Figure 1g), respectively.

S2.3 Time-resolved emission spectrum

The raw (not temporally broadened) emission spectrum was obtained by:

$$\sigma_{\mathrm{raw}}(E,t) = \sum_{n}^{n_{\mathrm{traj}}} (f_{\mathrm{osc}})_n \mathrm{e}^{-\frac{(E-E_n)^2}{W}}, \tag{3}$$

where E_n and $(f_{\text{osc}})_n$ are the energy difference and oscillator strength for the active→ground state transition and $W = \frac{(0.25\,\text{eV})^2}{4\ln 2}$ defines the width of the Gaussians used to broaden the spectrum on the energy axis (0.25 eV FWHM). To obtain the temporally broadened emission spectrum, the raw spectrum was additionally convoluted in the time domain with a Gaussian:

$$G(t) = \mathrm{e}^{-t^2/\beta}, \tag{4}$$

with $\beta = \frac{(100\,\text{fs})^2}{4\ln 2}$ defining the temporal width (100 fs FWHM).

In order to obtain an estimate of the luminescence decay time, the broadened emission spectrum was integrated over all energies and fitted with a bi-exponential decay function $f(t)$ convoluted with $G(t)$. The bi-exponential fitting function is:

$$f(t) = A\left(r\mathrm{e}^{-\frac{t}{\tau_1}} + (1-r)\mathrm{e}^{-\frac{t}{\tau_2}}\right). \tag{5}$$

Here, A is an overall intensity factor, τ_1 is the fast time constant, τ_2 is the slow time constant, and r is the ratio of the contributions of fast and slow channel.

S2.4 Charge-transfer character analysis

In order to follow the evolution of the electronic wave function, at each time step we computed charge transfer descriptors for all excited states with the TheoDORE program.[30,31] For the analysis, we divided the complex into 9 fragments: (1) Re, (2)–(4) the three CO ligands, (5) imidazole N atoms, (6) imidazole C atoms, (7) phenanthroline N atoms and C atoms in para-position to the N atoms, (8) all other phenanthroline C atoms except for the C=C bridge, and (9) the C=C bridge. H atoms were always included in the fragment of their parent atom. See Supplementary Fig. S3 for a graphical depiction. This fragmentation scheme was chosen based on a correlation analysis as described elsewhere.[31]

From the charge transfer analysis, for each of the 94 trajectories for each of the 500 time steps and for each of the 31 states, one obtains a 9×9 matrix with elements Ω_{AB} that describes how much contribution of the wave function is given by charge transfer between fragments A and B. From this data, we first compute the charge transfer matrix for the total electronic wave function for that trajectory and step:

$$\Omega_{AB}^{\text{traj}}(t) = \sum_i \left|\sum_\alpha U_{i\alpha}(t) c_\alpha^{\text{diag}}(t)\right|^2 \Omega_{AB}^i(t), \tag{6}$$

where Ω_{AB}^i is an element of the charge transfer matrix for MCH state i, and the "traj" superscript of $U_{i\alpha}$, c_α^{diag}, and $\Omega_{AB}^i(t)$ was omitted for clarity.

After finding the charge transfer matrices for the total electronic wave function of each trajectory $\Omega_{AB}^{\text{traj}}(t)$, we average them across all trajectories, leaving one 9×9 matrix for each time step:

$$\Omega_{AB}^{\text{total}}(t) = \frac{1}{N_{\text{traj}}} \sum_{\text{traj}} \Omega_{AB}^{\text{traj}}(t). \tag{7}$$

For a qualitative overview over the electronic character, we sum up multiple matrix elements:

$$\Omega_{\text{Re(CO)}_3 \to \text{phen}}(t) = \sum_{A \in \text{Re, CO}} \sum_{B \in \text{phen}} \Omega_{AB}^{\text{total}}(t), \tag{8}$$

$$\Omega_{\text{phen} \to \text{phen}}(t) = \sum_{A \in \text{phen}} \sum_{B \in \text{phen}} \Omega_{AB}^{\text{total}}(t), \tag{9}$$

$$\Omega_{\text{im} \to \text{phen}}(t) = \sum_{A \in \text{im}} \sum_{B \in \text{phen}} \Omega_{AB}^{\text{total}}(t). \tag{10}$$

Note that the sum of all elements of $\Omega^{\text{total}}(t)$ is equal to one.

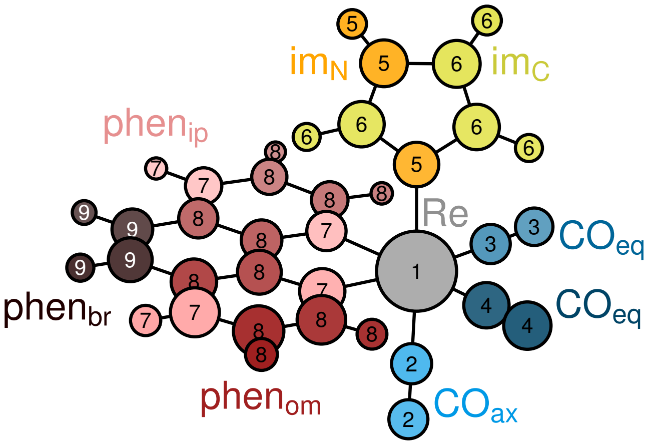

Figure S3: Fragmentation scheme for charge transfer analysis. For details on the charge transfer analysis see Supplementary Section S2.4.

S3 Supplementary results: Perturbational spin-orbit couplings

In $[Re(CO)_3(Im)(Phen)]^+$ the magnitude of the spin–orbit couplings (SOCs) are very large (several hundred cm^{-1} for some matrix elements), especially in relation to the small energy gaps. The most accurate energies of the mixed spin–orbit states are therefore obtained via a variational 2-component treatment,[32] where the full spin–orbit Hamiltonian is diagonalized in the full space spanned by all possible TDA excitations. However, in the SHARC dynamics simulations, we employ a perturbational approach,[23,28] where in a first step the spin-free Hamiltonian is diagonalized in the full excitation space, yielding a number of singlet and triplet states. In the second step, the spin–orbit Hamiltonian is constructed in the subspace spanned by these singlet and triplet states, and diagonalized to yield the spin–orbit energies. This approach neglects the spin–orbit couplings between the subspace and all higher-lying states, hence the spin–orbit analogue of "dynamical correlation energy" is neglected in the perturbational treatment.

In Supplementary Fig. S4 we show whether the perturbational spin–orbit treatment is sufficient and hence whether the SHARC approach is safely applicable to $[Re(CO)_3(Im)(Phen)]^+$. The figure compares the energies of the few lowest states of $[Re(CO)_3(Im)(Phen)]^+$ obtained with different approaches: (a) spin-free excitation energies (i.e., from step 1 of the perturbational treatment), (b,c,d) spin–orbit excitation energies from the perturbational approach, using different amounts of singlet and triplet states, and (e) the variational 2-component treatment. The excitation energies were computed with the above-described method, for one randomly chosen initial condition geometry of $[Re(CO)_3(Im)(Phen)]^+$. In the case that the number of states in the subspace is equal to the full excitation space, then the perturbational approach should deliver the same energies as the variational approach. As the full space contains 43,788 excitations with the used basis set, obviously only a small fraction of the excitation space can be used in the perturbational approach. However, as can be seen, the perturbational approach delivers excellent excitation energies when compared to the 2-component calculation. The following RMSDs (relative to the variational calculation) for the excitation energies were obtained: (a) 72 meV, (b) 3.8 meV, (c) 3.5 meV, (d) 3.2 meV (strictly decreasing in accord with the variational principle). This shows that even with the minimal number of singlet and triplet states, the perturbational approach can be used to reliably compute spin–orbit energies for third-row transition metal complexes. The results also show that it is not efficient to go beyond the minimal number of states, because the small improvement in accuracy does not merit the expense of computing the extra singlets and triplets. However, one can also clearly recognize that the spin-free energies are not reproducing the energy gaps well.

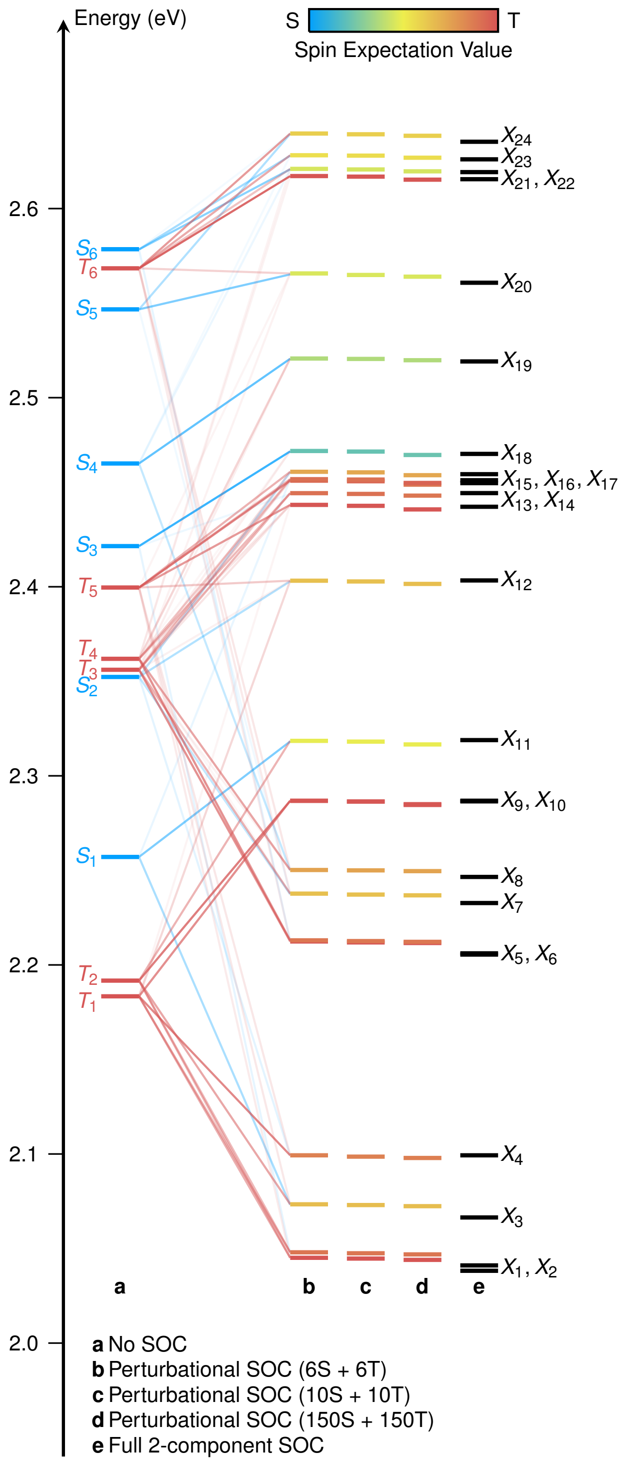

Figure S4: Comparison of energies of spin–orbit coupled states using different relativistic approximations. These approximations are: **a** no SOCs, **b** perturbational SOC for 6 singlets and 6 triplets, **c** for 10 singlets and 10 triplets, **d** for 150 singlets and 150 triplets, and **e** for full variational 2-component SOC (equivalent to 43,788 singlets and 43,788 triplets).

S4 Supplementary results: Evolution of electronic populations

Supplementary Figure S5 shows the same population data as Figure 1 in the main manuscript, but as a regular line plot.

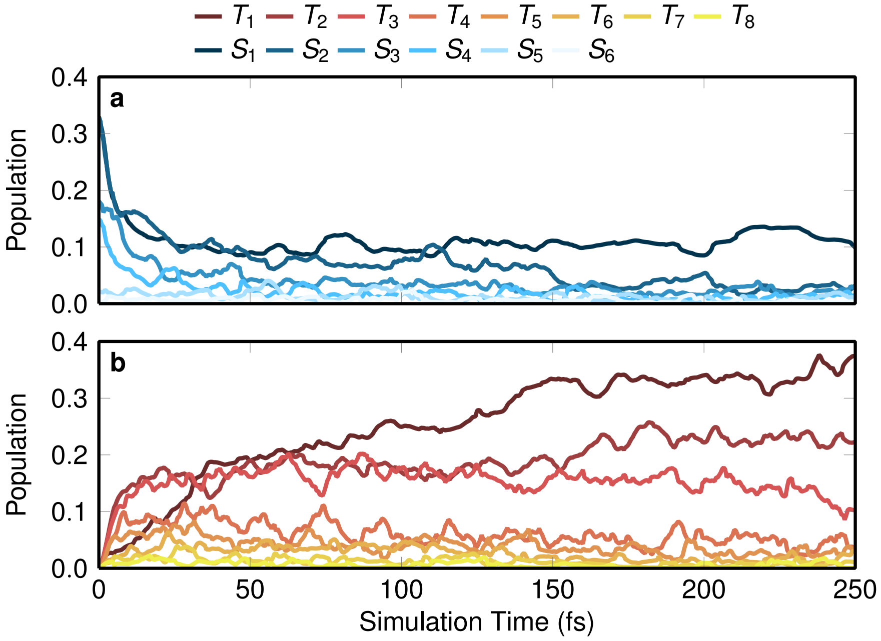

Figure S5: Line plot of the temporal evolution of the electronic populations. This figure shows the same population data as the stacked area plot in Figure 1 in the main text. **a** Singlet populations, **b** triplet populations. The population data was obtained from from 94 SHARC trajectories at the TD-B3LYP/T-DZP level of theory.

S5 Supplementary results: Definition of the spin–orbit wave packet

This section provides a formal definition of the "spin–orbit wave packet" that is introduced in the main manuscript.

We begin with the definition of the full electronic Hamiltonian:

$$\hat{H}^{\text{full}} = \hat{H}^{\text{spin-free}} + \hat{H}^{\text{SOC}}, \tag{11}$$

that was already mentioned in passing in Section S3. Importantly, one can either obtain the set of eigenfunctions of the spin-free Hamiltonian:

$$\hat{H}^{\text{spin-free}}|\psi_\alpha^{\text{spin-free}}\rangle = E_\alpha^{\text{spin-free}}|\psi_\alpha^{\text{spin-free}}\rangle \tag{12}$$

or the set of eigenfunctions of the full Hamiltonian:

$$\hat{H}^{\text{full}}|\phi_i^{\text{full}}\rangle = E_i^{\text{full}}|\phi_i^{\text{full}}\rangle. \tag{13}$$

Note that the eigenfunctions of the latter Hamiltonian do in general not have a well-defined spin multiplicity. Therefore, it is possible to represent the eigenfunctions of the full Hamiltonian as linear combinations of the spin-free eigenfunctions:

$$|\phi_i^{\text{full}}\rangle = \sum_\alpha U_{\alpha i}|\psi_\alpha^{\text{spin-free}}\rangle. \tag{14}$$

Equally, the spin-free eigenfunctions can be represented as linear combination of the full eigenfunctions:

$$|\psi_\alpha^{\text{spin-free}}\rangle = \sum_i U_{\alpha i}|\phi_i^{\text{full}}\rangle. \tag{15}$$

Hence, the eigenfunctions of the full Hamiltonian can conveniently be called "spin-mixed". As an example, the states X_1 to X_{24} in Figure S4 are spin-mixed eigenstates of the Hamiltonian of $[\text{Re(CO)}_3\text{(Im)(Phen)}]^+$ at a particular nuclear geometry. The associated energies of these states are the spin-mixed eigenenergies E_i^{full}.

Now we can write the definition of a time-dependent electronic wave function, expanded in the basis of the spin-free eigenfunctions:

$$|\Psi(t)\rangle = \sum_\alpha c_\alpha^{\text{spin-free}}(t)|\psi_\alpha^{\text{spin-free}}\rangle. \tag{16}$$

Inserting this expansion into the time-dependent Schrödinger equation and premultiplying with $\langle\psi_\beta^{\text{spin-free}}|$ yields the equation of motion:

$$\frac{\partial c_\beta^{\text{spin-free}}}{\partial t} = -\frac{\mathrm{i}}{\hbar}\sum_\alpha \langle\psi_\beta^{\text{spin-free}}|\hat{H}^{\text{full}}|\psi_\alpha^{\text{spin-free}}\rangle c_\alpha^{\text{spin-free}}(t). \tag{17}$$

Unfortunately, the matrix elements $\langle\psi_\beta^{\text{spin-free}}|\hat{H}^{\text{full}}|\psi_\alpha^{\text{spin-free}}\rangle$ do not vanish for $\alpha \neq \beta$, so that the differential equation is not trivially solvable.

Alternatively, one can write the time-dependent electronic wave function in the basis of the eigenfunctions of the full Hamiltonian:

$$|\Psi(t)\rangle = \sum_i c_i^{\text{full}}(t)|\phi_i^{\text{full}}\rangle. \tag{18}$$

In the same way as above, one obtains the equation of motion:

$$\frac{\partial c_j^{\text{full}}}{\partial t} = -\frac{\mathrm{i}}{\hbar} \sum_i \langle \phi_j^{\text{full}} | \hat{H}^{\text{full}} | \phi_i^{\text{full}} \rangle c_i^{\text{full}}(t), \tag{19}$$

but here the Hamiltonian matrix is diagonal, so we can simplify to:

$$\frac{\partial c_j^{\text{full}}}{\partial t} = -\frac{\mathrm{i}}{\hbar} E_j^{\text{full}} c_j^{\text{full}}(t), \tag{20}$$

with E_j^{full} being the eigenenergies of the full Hamiltonian.

The last differential equation can easily be solved to yield the solution of the time-dependent electronic wave function:

$$|\Psi(t)\rangle = \sum_i c_i^{\text{full}}(0) \mathrm{e}^{-\frac{\mathrm{i}}{\hbar} E_j^{\text{full}} t} |\phi_i^{\text{full}}\rangle. \tag{21}$$

According to this derivation, the time evolution of the electronic wave function after excitation can best be described as a linear superposition of spin-mixed eigenstates of the full Hamiltonian including SOC. In this basis, only the complex phase of the expansion coefficients depend on time.

The expansion coefficients $c_i^{\text{full}}(0)$ are determined by projecting the initial wave function $|\Psi(0)\rangle$ on the spin-mixed eigenstates:

$$c_i^{\text{full}}(0) = \langle \Psi(0) | \phi_i^{\text{full}} \rangle. \tag{22}$$

This can be done by inserting the resolution of identity for the spin-free states:

$$c_i^{\text{full}}(0) = \sum_\alpha \underbrace{\langle \Psi(0) | \psi_\alpha^{\text{spin-free}} \rangle}_{c_\alpha^{\text{spin-free}}(0)} \underbrace{\langle \psi_\alpha^{\text{spin-free}} | \phi_i^{\text{full}} \rangle}_{U_{\alpha i}}. \tag{23}$$

Here we assume that the initial electronic wave function is purely singlet—assuming instantaneous, vertical excitation from the singlet ground state. In the simplest such case, $c_\alpha^{\text{spin-free}} = \delta_{\alpha\sigma}$, where σ is any single singlet state. Even in this case, multiple $c_i^{\text{full}}(0)$ will be non-zero. Hence, vertical excitation indeed prepares a coherent linear superposition of the eigenstates of the full Hamiltonian, making the term "spin–orbit wave packet" appropriate.

S6 Supplementary results: Non-Kasha behavior in population flux

The biexponential ISC process is a clear example of non-Kasha behavior. Population transfer from singlet to triplet occurs faster than internal conversion. To illustrate this point, Supplementary Fig. S6 presents the number of net hops between the different electronic states. As can be seen, the number of hops from the S_1 to the triplet states (18 in total) is much smaller than the number of hops from higher singlets to triplets (55 in total). Although the ratio of the net hops shown in Supplementary Fig. S6 are probably not statistically converged (due to including only 94 trajectories), the figure clearly shows the strong non-Kasha behavior of the system.

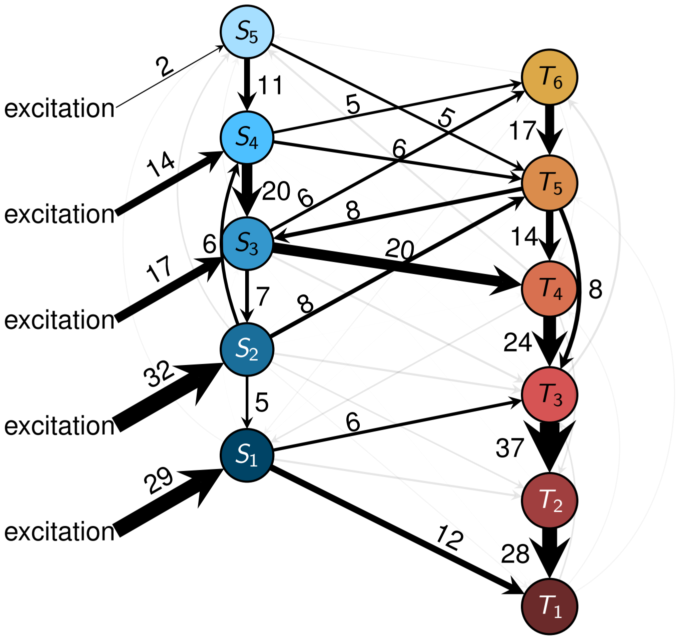

Figure S6: Number of net hops for all trajectories between the electronic states. The states S_0, S_6, T_7, T_8, which are included in the simulations but are never significantly populated, and labels for ≤ 4 hops are not shown for clarity.

S7 Supplementary results: Luminescence decay

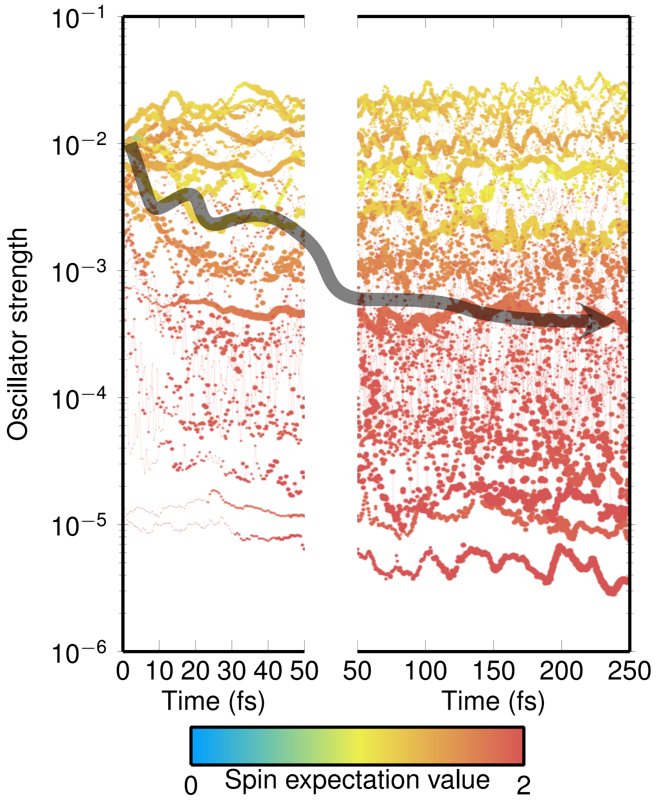

Figure S7: Scatter plot showing the relations between time, oscillator strength, spin expectation value, and state population. Time is given by the horizontal axis, oscillator strength by the vertical logarithmic axis, spin expectation value by color (singlet in blue, triplet in red, mixed states in intermediate shades), and state population by dot width. The data shown corresponds to the values averaged over all 94 trajectories. The arrow simplistically indicates the trend visible in the oscillator strength of the most populated states.

The luminescence decay that is observed experimentally and in the simulated emission spectrum is due to a complex interplay of different effects: (i) ISC from predominantly singlet states to predominantly triplet states, (ii) IC from bright singlet states to less bright singlet states, (iii) modification of brightness of the singlet states by nuclear motion. Additionally, the strong spin–orbit couplings induce strong mixing of the states. Supplementary Fig. S7 summarizes these different effects into one graph. It shows the temporal evolution of the average oscillator strength of each spin-mixed (diagonal) state on the vertical axis. From the vertical axis, one can read how the oscillator strength of the states changes due to the nuclear evolution. Additionally, the figure shows the spin expectation value as color code, and it indicates the most populated states by dot width.

According to the figure, at $t = 0$ all populated states (large dots) have a relatively large oscillator strength around 0.01. According to the color code, these states are already spin-mixed (spin expectation value around 1), but note that the total electronic wave function at $t = 0$ is a pure singlet, because the total wave function is a linear combination of the spin-mixed states. Over the course of the simulation, the oscillator strength of some of the strongly populated states decreases (e.g., between 20 and 30 fs from 0.004 to 0.002. More importantly, ISC drives population from the predominantly-singlet states that show high oscillator strengths to the predominantly-triplet states that have lower oscillator strength. Most relevant in this respect is the depopulation of the brightest states (oscillator strength above 0.01), which are largely dominated by S_3 and higher singlet states.

Supplementary References

[1] S. Mai, H. Gattuso, A. Monari and L. González, *Front. Chem.*, 2018, **6**, 495.

[2] D. A. Case, D. S. Cerutti, T. E. Cheatham, III, T. A. Darden, R. E. Duke, T. J. Giese, H. Gohlke, A. W. Goetz, D. Greene, N. Homeyer, S. Izadi, A. Kovalenko, T. S. Lee, S. LeGrand, P. Li, C. Lin, J. Liu, T. Luchko, R. Luo, D. Mermelstein, K. M. Merz, G. Monard, H. Nguyen, I. Omelyan, A. Onufriev, F. Pan, R. Qi, D. R. Roe, A. Roitberg, C. Sagui, C. L. Simmerling, W. M. Botello-Smith, J. Swails, R. C. Walker, J. Wang, R. M. Wolf, X. Wu, L. Xiao, D. M. York and P. A. Kollman, *AMBER 2017*, http://ambermd.org/, 2017.

[3] E. J. Baerends, T. Ziegler, A. J. Atkins, J. Autschbach, D. Bashford, O. Baseggio, A. Bérces, F. M. Bickelhaupt, C. Bo, P. M. Boerritger, L. Cavallo, C. Daul, D. P. Chong, D. V. Chulhai, L. Deng, R. M. Dickson, J. M. Dieterich, D. E. Ellis, M. van Faassen, A. Ghysels, A. Giammona, S. J. A. van Gisbergen, A. Goez, A. W. Götz, S. Gusarov, F. E. Harris, P. van den Hoek, Z. Hu, C. R. Jacob, H. Jacobsen, L. Jensen, L. Joubert, J. W. Kaminski, G. van Kessel, C. König, F. Kootstra, A. Kovalenko, M. Krykunov, E. van Lenthe, D. A. McCormack, A. Michalak, M. Mitoraj, S. M. Morton, J. Neugebauer, V. P. Nicu, L. Noodleman, V. P. Osinga, S. Patchkovskii, M. Pavanello, C. A. Peeples, P. H. T. Philipsen, D. Post, C. C. Pye, H. Ramanantoanina, P. Ramos, W. Ravenek, J. I. Rodríguez, P. Ros, R. Rüger, P. R. T. Schipper, D. Schlüns, H. van Schoot, G. Schreckenbach, J. S. Seldenthuis, M. Seth, J. G. Snijders, M. Solà, S. M., M. Swart, D. Swerhone, G. te Velde, V. Tognetti, P. Vernooijs, L. Versluis, L. Visscher, O. Visser, F. Wang, T. A. Wesolowski, E. M. van Wezenbeek, G. Wiesenekker, S. K. Wolff, T. K. Woo and A. L. Yakovlev, *Amsterdam density functional modeling suite 2017*, SCM, Theoretical Chemistry, Vrije Universiteit, Amsterdam, The Netherlands, https://www.scm.com, 2017.

[4] S. Mai, M. Richter, M. Heindl, M. F. S. J. Menger, A. J. Atkins, M. Ruckenbauer, F. Plasser, M. Oppel, P. Marquetand and L. González, *SHARC2.0: Surface Hopping Including Arbitrary Couplings – Program Package for Non-Adiabatic Dynamics*, sharc-md.org, 2018.

[5] S. Mai, P. Marquetand and L. González, *WIREs Comput. Mol. Sci.*, 2018, **8**, e1370.

[6] A. Pérez, I. Marchán, D. Svozil, J. Sponer, T. E. Cheatham, C. A. Laughton and M. Orozco, *Biophys. J.*, 2007, **92**, 3817 – 3829.

[7] J. Wang, R. M. Wolf, J. W. Caldwell, P. A. Kollman and D. A. Case, *J. Comput. Chem.*, 2004, **25**, 1157–1174.

[8] C. I. Bayly, P. Cieplak, W. Cornell and P. A. Kollman, *J. Phys. Chem.*, 1993, **97**, 10269–10280.

[9] P. J. Hay and W. R. Wadt, *J. Chem. Phys.*, 1985, **82**, 270–283.

[10] M. J. Frisch, G. W. Trucks, H. B. Schlegel, G. E. Scuseria, M. A. Robb, J. R. Cheeseman, G. Scalmani, V. Barone, B. Mennucci, G. A. Petersson, H. Nakatsuji, M. Caricato, X. Li, H. P. Hratchian, A. F. Izmaylov, J. Bloino, G. Zheng, J. L. Sonnenberg, M. Hada, M. Ehara, K. Toyota, R. Fukuda, J. Hasegawa, M. Ishida, T. Nakajima, Y. Honda, O. Kitao, H. Nakai, T. Vreven, J. A. Montgomery, Jr., J. E. Peralta, F. Ogliaro, M. Bearpark, J. J. Heyd, E. Brothers, K. N. Kudin, V. N. Staroverov, R. Kobayashi, J. Normand, K. Raghavachari, A. Rendell, J. C. Burant, S. S. Iyengar, J. Tomasi, M. Cossi, N. Rega, J. M. Millam, M. Klene, J. E. Knox, J. B. Cross, V. Bakken, C. Adamo, J. Jaramillo, R. Gomperts, R. E. Stratmann, O. Yazyev, A. J. Austin, R. Cammi, C. Pomelli, J. W. Ochterski, R. L. Martin, K. Morokuma, V. G. Zakrzewski, G. A. Voth, P. Salvador, J. J. Dannenberg,

S. Dapprich, A. D. Daniels, Ö. Farkas, J. B. Foresman, J. V. Ortiz, J. Cioslowski, and D. J. Fox, *Gaussian 09, Revision A.1*, Gaussian, Inc.: Wallingford CT, 2009, 2009.

[11] B. L. Oliveira, I. S. Moreira, P. A. Fernandes, M. J. Ramos, I. Santos and J. D. G. Correia, *J. Mol. Graph. Model.*, 2013, **45**, 13 – 25.

[12] W. L. Jorgensen, J. Chandrasekhar, J. D. Madura, R. W. Impey and M. L. Klein, *J. Chem. Phys.*, 1983, **79**, 926–935.

[13] N. Klaffki, O. Weingart, M. Garavelli and E. Spohr, *Phys. Chem. Chem. Phys.*, 2012, **14**, 14299–14305.

[14] M. Barbatti and K. Sen, *Int. J. Quantum Chem.*, 2016, **116**, 762–771.

[15] J. P. Zobel, M. Heindl, J. J. Nogueira and L. González, *J. Chem. Theory Comput.*, 2018, DOI: 10.1021/acs.jctc.8b00198.

[16] A. El Nahhas, C. Consani, A. M. Blanco-Rodríguez, K. M. Lancaster, O. Braem, A. Cannizzo, M. Towrie, I. P. Clark, S. Záliš, M. Chergui and A. Vlček, *Inorg. Chem.*, 2011, **50**, 2932–2943.

[17] M. Barbatti, G. Granucci, M. Persico, M. Ruckenbauer, M. Vazdar, M. Eckert-Maksić and H. Lischka, *J. Photochem. Photobiol. A*, 2007, **190**, 228–240.

[18] A. D. Becke, *J. Chem. Phys.*, 1993, **98**, 5648–5652.

[19] S. Grimme, S. Ehrlich and L. Goerigk, *J. Comput. Chem.*, 2011, **32**, 1456–1465.

[20] E. van Lenthe, E. J. Baerends and J. G. Snijders, *J. Chem. Phys.*, 1993, **99**, 4597–4610.

[21] E. van Lenthe and E. J. Baerends, *J. Comput. Chem.*, 2003, **24**, 1142–1156.

[22] S. Mai, H. Gattuso, M. Fumanal, A. Muñoz Losa, A. Monari, C. Daniel and L. González, *Phys. Chem. Chem. Phys.*, 2017, **19**, 27240–27250.

[23] F. Wang and T. Ziegler, *J. Chem. Phys.*, 2005, **123**, 154102.

[24] G. Granucci, M. Persico and A. Toniolo, *J. Chem. Phys.*, 2001, **114**, 10608–10615.

[25] F. Plasser, M. Ruckenbauer, S. Mai, M. Oppel, P. Marquetand and L. González, *J. Chem. Theory Comput.*, 2016, **12**, 1207.

[26] A. J. Atkins and L. González, *J. Phys. Chem. Lett.*, 2017, **8**, 3840–3845.

[27] G. Granucci and M. Persico, *J. Chem. Phys.*, 2007, **126**, 134114.

[28] S. Mai, T. Müller, P. Marquetand, F. Plasser, H. Lischka and L. González, *J. Chem. Phys.*, 2014, **141**, 074105.

[29] B. R. Landry, M. J. Falk and J. E. Subotnik, *J. Chem. Phys.*, 2013, **139**, 211101.

[30] F. Plasser, *TheoDORE: A package for theoretical density, orbital relaxation, and exciton analysis*, http://theodore-qc.sourceforge.net, 2017.

[31] S. Mai, F. Plasser, J. Dorn, M. Fumanal, C. Daniel and L. González, *Coord. Chem. Rev.*, 2018, **361**, 74–97.

[32] F. Wang, T. Ziegler, E. van Lenthe, S. van Gisbergen and E. J. Baerends, *J. Chem. Phys.*, 2005, **122**, 204103.
